# Mental Pain and Suicide: A Systematic Review of the Literature

**DOI:** 10.3389/fpsyt.2016.00108

**Published:** 2016-06-20

**Authors:** Maria Cristina Verrocchio, Danilo Carrozzino, Daniela Marchetti, Kate Andreasson, Mario Fulcheri, Per Bech

**Affiliations:** ^1^Department of Psychological, Health, and Territorial Sciences, G. d’Annunzio University, Chieti, Italy; ^2^Psychiatric Research Unit, Mental Health Centre North Zealand, Copenhagen University Hospital, Hillerød, Denmark

**Keywords:** mental pain, psychological pain, psychache, suicidal ideation, suicide attempt, suicidal behavior

## Abstract

**Background:**

Mental pain, defined as a subjective experience characterized by perception of strong negative feelings and changes in the self and its function, is no less real than other types of grief. Mental pain has been considered to be a distinct entity from depression. We have performed a systematic review analyzing the relationship between mental pain and suicide by providing a qualitative data synthesis of the studies.

**Methods:**

We have conducted, in accordance with PRISMA guidelines, a systematic search for the literature in PubMed, Web Of Science, and Scopus. Search terms were “mental pain” “OR” “psychological pain” OR “psychache” combined with the Boolean “AND” operator with “suicid*.” In addition, a manual search of the literature, only including the term “psychache,” was performed on Google Scholar for further studies not yet identified.

**Results:**

Initial search identified 1450 citations. A total of 42 research reports met the predefined inclusion criteria and were analyzed. Mental pain was found to be a significant predictive factor of suicide risk, even in the absence of a diagnosed mental disorder. Specifically, mental pain is a stronger factor of vulnerability of suicidal ideation than depression.

**Conclusion:**

Mental pain is a core clinical factor for understanding suicide, both in the context of mood disorders and independently from depression. Health care professionals need to be aware of the higher suicidal risk in patients reporting mental pain. In this regard, psychological assessment should include a clinimetric evaluation of mental pain in order to further detect its contribution to suicidal tendency.

## Introduction

The World Health Organization estimated 804,000 suicide deaths occurred worldwide in 2012, representing an annual global age-standardized suicide rate of 11.4 per 100,000 population (15.0 for males and 8.0 for females). For every completed suicide, there are many more people who attempt suicide every year (1). The suicide is the consequence of a complex interaction of several variables, including psychological (i.e., personality traits, individual characteristics, emotional elements, and dysregulation), biological (i.e., genetics, medications, comorbid illness), and environmental factors (i.e., social support, demographics) (2). Despite each of risk factors proposed having some power in the prediction of suicide, a significant body of data (3) emphasized the prominent association between mental pain or psychache and suicidal behavior. By following the literature, mental pain, psychache, and psychological pain are specific terms used to refer to the same construct (4). In this regard, we have used the specific expression reported by authors referring to pain originating from an individual psychological dimension. In the “cubic model” defined by Shneidman (5), psychache is defined as one of three essential dimensions, when individuals are considering suicide. The cubic model is the conceptualization of suicidal behavior. The two other dimensions are stress and perturbation. Psychache, as the central aspect of suicidal behavior, provides a theoretical definition of this construct as a general psychological pain reaching intolerable intensity that encompasses shame, guilt, humiliation, loneliness, fear, angst, and dread (6). That is, other psychological factors (e.g., depression) are relevant only to the extent in that they relate to psychache (7) that acts as a mediator of other risk factors. In other words, suicide would not occur without psychological pain (8). Orbach et al. (9) have described nine dimensions of mental pain: lack of control, irreversibility of pain, emotional flooding, estrangement, emotional flooding, confusion, social distancing, and emptiness. Buchwald (10) has considered the suicide as “a permanent solution to a temporary problem” resulting from an overwhelming angst of the subject (i.e., psychache). When taking into consideration the evidence that there are, on the one hand, depressed patients who did not die by suicide, and, on the other hand, not clinically depressed suicide attempters, several authors have proposed that there is a core risk factor for suicide, which has been conceptualized as psychache or psychological pain (11). In this regard, a recent research report from Soumani et al. (12) highlighted that this type of mental pain contributes significantly to suicide risk independent of depression.

The clinical link between psychological pain and suicide, as well as the concept of psychache as essential factor affecting suicidality, has been established with empirical research studies showing psychache’s full mediation effects on the suicide risk (13). Furthermore, growing evidence has considered the suicide as a behavior motivated by the desire to escape from unbearable psychological pain (14–16). In a recent editorial, de Leon et al. (17) proposes that suicidal ideation, suicide attempts, and completed suicide may not be continuous phenomena and they can be influenced at different levels by the relative weight of psychosocial versus biological predictors. These authors argued that mental pain is the construct that unifies all suicide behavior even if how mental pain can be explained varies across suicide behavior levels. Studies on this issue are needed, by including also the evaluation of hopelessness that is an important factor for understanding the suicidal state of mind (18).

Despite the extensively reported data of the literature supporting the association between psychache and several dimensions of suicidality, such as suicide thoughts or ideation, suicide motivation and preparation, suicide attempt, and suicide act (19), to date, no systematic review study was fulfilled.

On this preliminary background, the aim of the current study is to provide a systematic review of original studies by focusing on the relationship (including associations or correlations, comparisons and differences, mediating roles, as well as contributions or predictions) between mental pain and the core suicidal clinical factors, namely ideation, attempt, and suicidal act.

## Materials and Methods

### Information Sources and Searches

In line with Preferred Reporting Items for Systematic Reviews and Meta-Analyses (PRISMA) guidelines (20), a comprehensive electronic search strategy was used to identify peer-reviewed articles on the relationship between mental pain and suicide up to May 2016. The following keywords were used: “mental pain OR psychological pain OR psychache” AND “suicid*.” After the initial search was performed, the studies were screened for eligibility; their relevance was assessed using at first their titles and abstracts, and finally the full review of papers. Searching and eligibility of target responses were carried out independently by two investigators; disagreements were resolved by consensus among these primary raters and a senior investigator. Electronic research-literature databases searched included PubMed, Web of Science, and Scopus. A manual search of the literature, only including the term “psychache,” was also performed on Google Scholar for further studies not yet identified. In order to detect any missed articles during the literature search, reference lists of candidate articles were reviewed, yielding no additional articles. For each excluded study, we determined which elements of the electronic search were not addressed.

### Eligibility Criteria

Papers were eligible for inclusion if they were original research reports in English language describing data on mental pain in relation to suicidal ideation, attempt, and behavior. We excluded peer-reviewed articles published prior to 1995, single case studies, reviews, meta-analyses, letters to the editor and commentaries, conference abstracts, books, and papers that were clearly irrelevant. Studies were discarded whether full text was not available. Results were not limited to chronological age of participants.

### Analysis and Data Synthesis

Due to the heterogeneity of study design, measures, and features of the samples, it was not possible to combine the results into a meta-analysis. Consequently, results have been described reporting data through a systematic review. Studies were categorized based on the sample recruited for the study, by summarizing and comparing significant information for each study.

## Results

**Figure 1:**
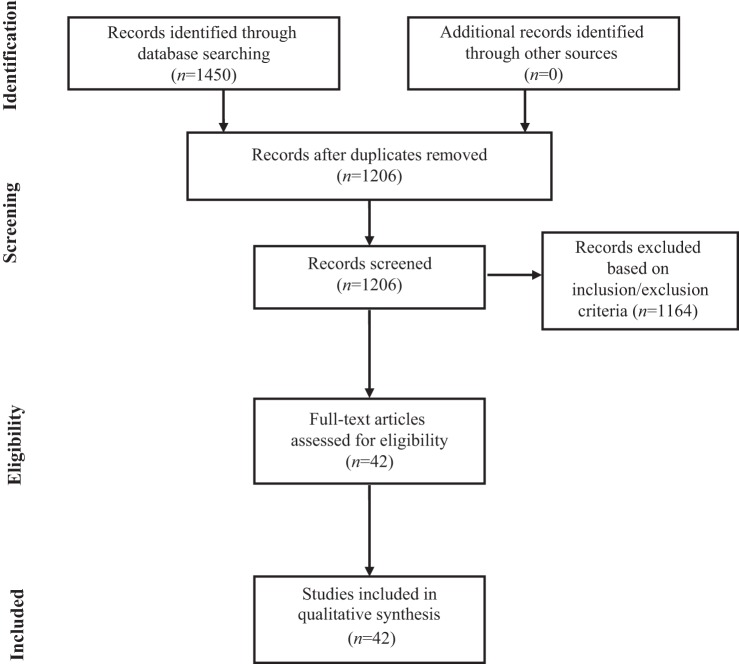
**Flowchart of the systematic search**.

The search of PubMed, Scopus, Web of Science, and Google Scholar databases provided a total of 1450 citations. Based both on inclusion and exclusion criteria, a total of 42 original research studies were identified and selected for inclusion in the systematic review, as reported in the flowchart displayed in Figure 1.

### Mental Pain and Suicide in Samples with Mood Disorders

Several studies conducted with mood disorder patients have shown significant associations between mental pain and suicidality (see Table 1 for a detailed description of reviewed studies). By focusing on a clinical sample consisting of patients with mood disorder (i.e., newly diagnosed depressed adult outpatients), Berlim et al. (21) reported a significant association between psychache and suicidality with a correlation identified as the highest in magnitude. These results were confirmed by Mee et al. (3), using a sample of 73 outpatients with major depression compared with 96 non-psychiatric controls. Xie et al. (16) have provided a further evidence supporting the relevance of psychological pain in the risk of suicide by demonstrating that outpatients with major depressive episodes and high levels of suicidal ideation showed anticipatory anhedonia and stronger pain avoidance matched to those with low levels of suicidal ideation and healthy controls.

**Table 1:** **Studies on mental pain and suicide in samples with mood disorders**.

Study	Title	Aim	Sample information	Measure of mental pain	Measure of suicide
Berlim et al. (21)	Psychache and suicidality in adult mood disordered outpatients in Brazil	To assess the prediction that psychache (as derived by psychological quality of life) is associated with suicidality over and above depressive symptoms, hopelessness, interpersonal, and physical domains of quality of life	Sample size: 60 diagnosed depressed adult patients Mean age: 49.6 (SD 12.41) years Female: 83%	WHO-QOL psychological domain (6 items)	BDI suicidality item; MINI questions about suicidality
Caceda et al. (23)	Impulsive choice and psychological pain in acutely suicidal depressed patients	To examine the relationship of psychological pain and choice impulsivity with acute suicidal behavior	Sample size: 82 participants divided into four groups; G1, suicide attempters (20); G2, suicide ideators (22); G3, depressed controls (20); G4, healthy controls (20) Mean age: G1 = 36.4 (SD = 3.8) years, G2 = 43.1 (SD = 2.7) years, G3 = 46.2 (SD = 2.5) years, G4 = 39.3 (SD = 4.5) years Female: G1 = 60%, G2 = 50%, G3 = 65%, G4 = 50%	PAS	C-SSRS BDI
Huanhuan et al. (24)	Clarifying the role of psychological pain in the risks of suicidal ideation and suicidal acts among patients with major depressive episodes	To further investigate the role of psychological pain in suicidal ideation and suicidal acts among patients with major depressive episodes	Sample size: 111 depressed patients divided into two groups; G1, depressed patients with a history of suicide attempts (28); G2, depressed patients with no history of suicide attempts (83) Mean age: G1 = 28.93 (SD = 12.37), G2 = 33.99 (SD = 12.63) Female: G1 = 71%, G2 = 60%	TDPPS PAS	BSS Structured clinical interview assessing suicidal acts that include some degree of seriousness and/or lethality
Mee et al. (3)	Assessment of psychological pain in major depressive episodes	To evaluate, developing a brief measure of psychological pain, the role of psychological pain in suicide, and depression	Sample size: 168 participants divided into two groups; G1, major depressive episode patients (73); G2, non-psychiatric controls (95) Mean age: G1 males = 54.00 (SD = 12) and females = 46.00 (SD = 17); G2 males = 55.00 (SD = 17) and females = 45.00 (SD = 17) Female: G1 = 38%, G2 = 40%	MBPPAS	SBQ
Meerwijk and Weiss (26)	Does suicidal desire moderate the association between frontal delta power and psychological pain?	To investigate the moderating effect of recent suicidal desire on the association between resting-state neurophysiological parameters and psychological pain	Sample size: 35 adults with a history of depression, divided into two groups Mean age: 34.91 (SD = 11.60) Female: 77%	PAS OMMP	BSS
Olié et al. (22)	Higher psychological pain during a major depressive episode may be a factor of vulnerability to suicidal ideation and act	To test the hypothesis that higher psychological pain during a major depressive episode may represent a trait of vulnerability to suicidal ideation and suicidal acts	Sample size: 210 patients divided into three groups; G1, recent suicide attempters (87); G2, former suicide attempters (61); G3, non-suicide attempters (62) Mean age: G1 = 40 (NA) years, G2 = 38 (NA) years, G3 = 38 (NA) years Female: G1 = 67%, G2 = 74%, G3 = 73%	Three items assessing intensity of psychological pain currently and during the last 15 days (including usual and maximum)	Two items measuring intensity of suicidal ideation and frequency of suicidal ideation
van Heeringen et al. (25)	The functional neuroanatomy of mental pain in depression	To evaluate neurofunctional aspects of mental pain related to suicidality	Sample size: 39 depressed patients divided into three groups; G1, 13 patients with low levels of mental pain; G2, 13 patients with medium levels of mental pain; G3, 13 patients with high levels of mental pain Mean age: G1 = 42.5 (SD = 15.0) years, G2 = 35.15 (SD = 12.9) years, G3 = 44.77 (SD = 15.3) years Female: G1 = 53.8%, G2 = 61.5%, G3 = 53.8%	OMMP	Item 9 of the BDI assessing occurrence and severity of suicidal ideation
Xie et al. (16)	Anhedonia and pain avoidance in the suicidal mind: behavioral evidence for motivational manifestations of suicidal ideation in patients with major depressive disorder	To provide empirical evidence for the relationship between anhedonia, psychological pain (i.e., pain avoidance), and suicidal ideation	Sample size: 60 participants divided into three groups; G1, high suicide ideation participants (27 depressed patients); G2, low suicide ideation participants (13 depressed patients); G3 healthy control participants (20) Mean age: G1 = 14.07 (SD = 3.03), G2 = 13.77 (SD = 3.39), G3 = 14.75 (SD = 2.83) Female: G1 = 70.4%, G2 = 69.2%, G3 = 60.0%	TDPPS PAS	BSS

*NA, Not Available; G1, Group 1; G2, Group 2; G3, Group 3; G4, Group 4*.

*C-SSRS, Columbia Suicide Severity Rating Scale; BDI, Beck Depression Inventory; BSS, Beck Scale for Suicide Ideation; MBPPAS, Mee–Bunney Psychological Pain Assessment Scale; MINI, Mini International Neuropsychiatric Interview; OMMP, Orbach and Mikulincer Mental Pain Scale; PAS, Psychache Scale; SBQ, Suicidal Behavior Questionnaire; TDPPS, three-dimensional Psychological Pain Scale; TMPS, Tolerance for Mental Pain Scale; WHO-QOL, World Health Organization’s Quality of Life Instrument*.

High levels of psychache during a major depression may represent a condition of vulnerability for suicidal ideation and act. Olié et al. (22), by comparing 87 recent suicide attempters both with 61 patients with a past history of suicidal acts and 62 non-attempters patients, have found that higher current psychological pain was more frequent in recent and former suicide attempters than in non-attempters. Moreover, the authors also showed that the severity of current psychological pain was significantly associated with the intensity [OR = 2.7 (95% CI: 1.5–4.9)] and frequency of suicidal ideation [OR = 3.0 (95% CI: 1.7–5.5)]. These data further confirm that a higher tendency to experience current mental pain during a major depression is a significant trait of vulnerability to suicidal behavior (i.e., ideation and acts), differentiating patients with a past or current history of suicide attempts from patients without any suicidal history. Caceda et al. (23), evaluating a sample of 62 depressed patients compared with a sample of 20 healthy controls, have highlighted that psychological pain predicted the presence of suicidal ideation in the overall sample by differentiating, using logistic regression analysis, between the suicide attempt and suicidal ideation groups. In a study with 111 outpatients with major depression, Huanhuan et al. (24), by using the stepwise regression analyses, were able to demonstrate that pain avoidance (i.e., the desire of escaping from unbearable psychological pain) was the only significant predictor of suicidal ideation at one’s worst point and of suicidal acts.

In order to demonstrate the association between the degree of mental pain and changes in the cerebral blood flow in specific areas of the brain (i.e., right occipital cortex, left inferior temporal gyrus, right dorsolateral prefrontal cortex, and right inferior frontal gyrus, as well as left medulla at pontine levels) van Heeringen et al. (25), by performing functional neuroimaging in 39 depressed inpatients, showed that scores of mental pain on the Orbach & Mikulincer Mental Pain Scale (9) correlated significantly with the scores on the Beck Depression Inventory suicidality item. A recent study by Meerwijk and Weiss (26) evidenced that recent suicide desire moderates the relationship between psychache and resting-state neurophysiological parameters. Decreased low-frequency heart rate variability and EEG delta power were interpreted by authors as indicators of less effective emotion regulations, including increased rumination and an inability to reappraise the causes and consequences of psychological pain.

### Mental Pain and Suicide in Different Clinical Samples

Studies conducted with different clinical groups (e.g., suicidal patients and/or psychiatric patients) showed significant associations between mental pain and suicide (27, 28) (Table 2). Orbach et al. (29) have found that suicidal patients scored showed significantly higher scores on mental pain rates than both psychiatric patients and control participants, with three specific mental pain factors (i.e., irreversibility, loss of control, and emptiness) having a unique contribution for the differentiation between suicidal and non-suicidal groups. These results, confirmed by Pompili et al. (30), have demonstrated that patients currently at risk for suicide showed significantly higher current psychache and higher worst-ever psychological pain.

**Table 2:** **Studies on mental pain and suicide in different clinical samples**.

Study	Title	Aim	Sample information	Measure of mental pain	Measure of suicide
Barak and Miron (27)	Writing characteristics of suicidal people on the internet: a psychological investigation of emerging social environments	To support, in Study 3, Shneidman’s original argument that there are specific themes that characterized suicidal people, such as unbearable emotional pain (and cognitive constriction), focusing on the content of online writers’ messages	Sample size: 64 online messages by 39 participants in the SAHAR suicidal support forum and by 24 participants in the sexual assault forum Mean age: NA Female: NA	Leenaars’ (1996) thematic guide for predicting suicide	NA
Campos and Holden (39)	Testing models relating rejection, depression, interpersonal needs, and psychache to suicide risk in non-clinical individuals	To evaluate a model of suicide risk based on the contribution of four psychological variables, parental rejection, depression, interpersonal need, and psychache	Sample size: 203 non-clinical participants Mean age: 37.86 (SD = 11.68) Female: 51%	PAS	SBQ-R
Gould et al. (38)	An evaluation of crisis hotline outcomes part 2: suicidal callers	To determine, among other objectives, predictors (i.e., intent to die, psychological pain, hopelessness) of suicidality after the call to crisis services/hotlines	Sample size: 1085 suicidal callers Mean age: NA Female: 61%	Two items assessing psychological pain	Nine questions about suicidal thoughts, plans, and attempts
Gvion et al. (36)	A proposed model of the development of suicidal ideations	To develop a model of suicide ideation in psychiatric patients and the general population taking into account the role of mental pain domain, aggressive-impulsive domain, communication difficulties domain, and life events	Sample size: 196 participants divided into three groups; G1, suicide attempters (92 psychiatric patients); G2, non-attempters (47 psychiatric patients); G3 controls (57 healthy subjects) Mean age: G1 = 38.93 (SD = 13.56) years, G2 = 40.96 (SD = 14.07) years, G3 = 37.28 (SD = 12.34) Female: G1 = 35%, G2 = 30%, G3 = 46%	OMMP	Item 9 of the BDI LRS
Gvion et al. (2)	Aggression–impulsivity, mental pain, and communication difficulties in medically serious and medically non-serious suicide attempters	To evaluate, among other objectives, the role of mental pain, depression, and hopelessness in differentiating suicide attempters from non-attempters	Sample size: 196 participants divided into four groups; G1, medically serious suicide attempters (43); G2, medically non-serious suicide attempters (49); G3, psychiatric control group (47); G4, healthy control group (57) Mean age: G1 = 37.37 (SD = 13.31) years, G2 = 40.31 (SD = 13.76) years, G3 = 40.96 (SD = 14.07), G4 = 37.28 (SD = 12.34) Female: G1 = 40%, G2 = 31%, G3 = 30%, G4 = 46%	OMMP	LRS Objective Planning Subscale of the SIS
Horesh et al. (34)	Medically serious versus non-serious suicide attempts: relationships of lethality and intent to clinical and interpersonal characteristics	To investigate, among other objectives, the relationship between mental pain and subjective/objective suicide intent in both medically serious and medically non-serious attempters	Sample size: 102 participants divided into two groups; G1, patients after a medically serious suicide attempt (35); G2, patients after a medically non-serious suicide attempt (67) Mean age: G1 = 39.70 (SD = 15.30) years, G2 = 37.30 (SD = 14.00) years Female: G1 = 49%; G2 = 54%	OMMP	SIS LRS
Leenars et al. (28)	Suicide notes in alcoholism	To assess whether suicide notes of alcoholics differ from suicide notes of non-alcoholics in Leenars’ dimensions of suicide, including unbearable pain	Sample size: 16 suicide notes of alcoholics and matched suicide notes of non-alcoholics Mean age: NA Female: NA	Suicide notes	Suicide notes
Levi et al. (32)	Mental pain and its communication in medically serious suicide attempts: an “impossible situation”	To test the hypothesis that mental pain is a general risk factor for suicidal behavior (and communication difficulties are a particular risk factor for medically serious suicidal behavior)	Sample size: 173 subjects divided into three groups; G1, patients after a medically serious suicide attempt (35); G2, patients after a medically non-serious suicide attempt (67); G3 healthy controls (71) Mean age: G1 = 39.70 (SD = 15.30) years; G2 = 37.30 (SD = 14.00) years; G3 = 36.50 (SD = 14.00) years Female: G1 = 49%; G2 = 54%; G3 = 48%	OMMP	LRS
Levi-Belz et al. (35)	Attachment patterns in medically serious suicide attempts: the mediating role of self-disclosure and loneliness	To assess the contribution of attachment style to medical lethality of the suicidal attempt above and beyond mental pain (and the meditational role of communication difficulties in the relationship between attachment style and medically serious suicide attempt)	Sample size: 102 patients divided into two groups; G1, patients after a medically serious suicide attempt (35); G2, patients after a medically non-serious suicide attempt (67) Mean age: G1 = 39.70 (SD = 15.30) years, G2 = 37.30 (SD = 14.00) years Female: G1 = 49%, G2 = 54%	Mental pain is only indirectly evaluated with measures of depression, hopelessness and negative life events, BDI, BHS, and LES, respectively	LRS SIS
Levi-Belz et al. (33)	Mental pain, communication difficulties, and medically serious suicide attempts: a case-control study	To assess the role of mental pain and communication difficulties in medically serious suicide attempt	Sample size: 336 participants divided into four groups; G1, medically serious suicide attempters (78); G2, medically non-serious suicide attempters (116); G3, psychiatric control group (47); G4, healthy control group (95) Mean age: G1 = 38.5 (SD = 14.2) years, G2 = 38.5 (SD = 13.9) years, G3 = 40.9 (SD = 14.0) years, G4 = 38.5 (SD = 14.2) years Female: G1 = 44%, G2 = 44%, G3 = 70%, G4 = 45%	OMMP	LRS
Levinger and Holden (41)	Reliability and validation of the Hebrew Version of the Reasons for Attempting Suicide Questionnaire (RASQ-H) and its importance for mental pain	To evaluate, among other objectives, relationships of the RASQ-H with mental pain and the tolerance of mental pain	Sample size: 97 participants divided into three groups; G1, suicide attempter inpatients (42); G2, non-suicidal psychiatric inpatients (26); G3, non-clinical individuals (29) Mean age: 19.51 (SD = 3.30); data for single groups NA Female: 50% of the total sample; rates for single groups NA	OMMP TMPS	RASQ-H BSS MAST LSAS
Levinger et al. (40)	The importance of mental pain and physical dissociation in youth suicidality	To assess whether physical dissociation can make a unique contribution to suicidal risk above and beyond the contributions of mental pain and low tolerance for that mental pain	Sample size: 123 young adults divided into three groups; G1, suicidal patients (42); G2, non-suicidal inpatients (36); G3 non-clinical group (45) Mean age: G1 = 18.60 (SD = 3.3) years, G2 = 21.08 (SD = 2.73) years, G3 = 19.29 (SD = 3.07) years Female: G1 = 55%, G2 = 42%, G3 = 56%	OMMP TMPS	MAST BSS LSAS
May et al. (31)	Descriptive and psychometric properties of the Inventory of Motivations for Suicide Attempts (IMSA) in an inpatient adolescent sample	To investigate, among other objectives, the motivations (e.g., psychache, hopelessness, and escape) adolescents endorsed for their suicide attempts	Sample size: 52 adolescent psychiatric inpatients who attempted suicide Mean age: 14.8 (SD = 1.4) Female: 85%	Psychache scale of the IMSA	Interview assessing lifetime suicide attempts C-SSRS
Nahaliel et al. (11)	Mental pain as a mediator of suicidal tendency: a path analysis	To examine the mediating role of mental pain in the relationship between number of lifetime losses, self-destruction, and suicidal tendency	Sample size: 150 adults divided into three groups; G1, suicide attempt patients (50); G2, non-suicidal psychiatric patients (50); G3, healthy controls (50) Mean age: G1 = 43.26 (SD = 14.5) years, G2 = 43.86 (SD = 15.4) years, G3 = 40.40 (SD = 16.1) years Female: G1 = 70%, G2 = 70%, G3 = 68%	OMMP	MAST
Orbach et al. (29)	Mental pain and its relationship to suicidality and life meaning	To test, among other objectives, Shneidman’s proposition – on the relationship between mental pain and suicide – by comparing the mental pain of suicidal and non-suicidal individuals	Sample size: 91 subjects divided into three groups; G1, suicide attempters patients (32); G2, non-suicide attempters patients (29); G3 control participants (30) Mean age: G1 = 32.43 (SD = 5.43) years, G2 = 34.28 (SD = 6.71) years, G3 = 31.62 (SD = 5.84) years Female: G1 = 56%, G2 = 62%, G3 = 53%	OMMP	MAST
Pompili et al. (30)	Psychache and suicide: a preliminary investigation	To explore the usefulness of Shneidman’s measure of psychache using a sample of psychiatric patients. one specific objective was to address the association between PPAS score and current suicidal risk and suicidal history	Sample size: 88 psychiatric patients Mean age: for males and females, 41.8 (SD = 14.0) years and 41.2 (SD = 14.1) years, respectively Female: 60%	PPAS	Section about suicidal risk of the MINI integrated with Clinician’s opinion
Reisch et al. (15)	An fMRI study on mental pain and suicidal behavior	To investigate the neural correlates of script-driven recall of mental pain plus suicide action	Sample size: 10 individuals who had attempted suicide 1 to 4 weeks prior to the interview Mean age: 38.5 (SD = 13.1) years Female: 100%	OMMP Mental pain sequences from narrative interviews	Suicide action and suicide attempt sequences from narrative interviews
Trakhtenbrot et al. (37)	Predictive value of psychological characteristics and suicide history on medical lethality of suicide attempts: a follow-up study of hospitalized patients	To test, among other assumptions, the hypothesis that mental pain, depression, and hopelessness are positively related to follow-up suicide attempt	Sample size: 153 subjects divided into three groups; G1, patients hospitalized for a medically serious suicide attempt (53); G2, patients hospitalized for a medically non-serious suicide attempt (64); G3, psychiatric control group (36) Mean age: G1 = 37.60 (SD = 12.25) years; G2 = 37.74 (SD = 13.05) years; G3 = 40.27 (SD = 13.26) years Female: G1 = 59%; G2 = 61%; G3 = 69%	OMMP	Clinician assessment of suicide attempts, medical severity of the attempts, and medical severity of the follow-up attempt

*NA, not available; G1, Group 1; G2, Group 2; G3, Group 3; G4, Group 4; S1, Study 1; S2, Study 2*.

*C-SSRS Columbia Suicide Severity Rating Scale; BDI Beck Depression Inventory; BHS Beck Hopelessness Scale; BSS Beck Scale for Suicide Ideation; IMSA Inventory of Motivations for Suicide Attempts; LES Life Event Scale; LRS Lethality Rating Scale; LSAS Lethality of Suicide Attempt Scale; MAST Multi-Attitude Suicidal Tendencies Scale; MBPPAS Mee–Bunney Psychological Pain Assessment Scale; MINI, Mini International Neuropsychiatric Interview; OMMP Orbach and Mikulincer Mental Pain Scale; PAS Psychache Scale; PPAS Psychological Pain Assessment Scale; RASQ-H Hebrew Reasons for Attempting Suicide Questionnaire; SBQ-R Suicidal Behavior Questionnaire-Revised; SIS Suicide Intent Scale; TMPS Tolerance for Mental Pain Scale*.

A recent research (31) on motivations for suicide attempts in an inpatient adolescent sample reported psychache, hopelessness, and escape as the three most strongly endorsed motivations.

Levi et al. (32) have found higher levels of unbearable psychological pain among clinical sample (i.e., medically serious suicide attempt) compared to healthy controls. Furthermore, by conducting a hierarchical regression analysis, the authors have demonstrated that the presence of suicidal behavior was significantly predicted by mental pain accounting for 48% of the variance. In this study, mental pain was a predictor of suicidal behavior, whereas the interpersonal and communication difficulties, such as low self-disclosure ability, in addition to schizoid traits, alexithymia, and loneliness, were predictors of the lethality and seriousness of suicidal behavior. In a more recent research, Levi-Belz et al. (33) showed that suicide attempters obtained significantly higher scores on individual experience of mental pain than controls (i.e., both non-suicidal psychiatric patients and healthy participants). Communication difficulties and mental pain play an important role in medically serious suicide attempts, but the contribution of each is different. Results have showed that mental pain differentiates suicide attempters from psychiatric and healthy controls, and only communication difficulties distinguished medically serious suicide attempters from medically non-serious suicide attempters. In addition, the interaction between mental pain and communication difficulties accounting for 23% of the variance in suicide lethality, above and beyond the contribution of each component alone. These findings showed that the severity of the attempt depends on the individual’s ability to communicate his or her distress to others. Gvion et al.(2), in a different sample (medically serious and medically non-serious suicide attempters), by performing a hierarchical regression analysis, demonstrated that mental pain factors only accounted for 3% of the variance and did not significantly predict suicide lethality. However, the interaction of mental pain and schizoid traits significantly predicted suicidal medical lethality after all other variables (i.e., aggression–impulsivity, and communication difficulties) had been entered. These findings suggest that the degree to which an individual can tolerate negative emotions (e.g., mental pain, violence, aggressive–impulsive tendencies, anger) may play an important role in the decision to attempt suicide.

In order to further study the lethality of the suicide intent, as dichotomously conceptualized in its subjective vs. objective components, Horesh et al. (34) have found that mental pain significantly contributed in the only prediction of the subjective intent having only a limited association with lethality of the suicidal attempt. By following another study on suicidal lethality, Levi-Belz et al. (35) have revealed that the attachment style significantly predicted (above and beyond the contribution of mental pain) the severity of the suicidal attempt. As regards the study of the suicide attempts, another research by Gvion et al. (36) provided a bidirectional model showing that hopelessness and depression mediated between mental pain and current suicidal ideation in suicide attempters. A recent follow-up study on psychiatric inpatients (37) demonstrated that mental pain did not predict a follow-up suicide attempt over time. Only hopelessness and depression were predictors of a follow-up attempt at suicide and the medical severity of the follow-up attempt.

The relationship between suicidal risk and mental pain was further evaluated in a study by Gould et al. (38) aimed at investigating the effectiveness of a telephone crisis services. Gould et al. (38) have found a significant reduction in suicide risk factors (i.e., plans, actions, and prior attempts) with diminishing levels of psychological pain from the beginning to the end of the call.

Other studies investigated the mediational role of mental pain. Nahaliel et al. (11) have showed that self-destruction causes both a direct effect on suicidal tendency, and an indirect effect mediated by the presence of mental pain. Subsequently, Campos and Holden (39) have revealed that psychache and interpersonal needs mediate the relationship between depression and suicide risk.

Mental pain seems to be a leading cause of suicide only when it is experienced as unbearable according to the cubic model of Shneidman (5). Recently, studies highlighted the clinical relevance of tolerance as a component of mental pain to explain its association with suicidality. In this regard, Levinger et al. (40), by using a multivariate analysis of covariance (i.e., MANCOVA), have demonstrated that suicidal respondents reported higher levels of mental pain, as well as a lower tolerance for such pain compared to non-suicidal group. Furthermore, when testing the hypothesis that mental pain and tolerance for this pain predict the suicidal risk, the authors confirmed that current mental pain was the strongest predictor of many aspects linked to suicidal behavior (i.e., self-reported suicidal ideation, suicide preparation, repulsion by life, and attraction to death). In addition, physical dissociation can increase the likelihood of choosing a suicidal act rather than another form of coping with intense and intolerable mental pain. Levinger et al. (40) suggested that when mental pain becomes intolerable and persistent, blocking awareness of the body and its signals renders the body a lifeless object and an easier target to attack.

A previous study from Levinger and Holden (41), when examining the reliability and validity of the Hebrew Version of the Reasons for Attempting Suicide Questionnaire (RASQ-H), has found that the correlations between psychache and its tolerance with the Internal Perturbation-Based Reasons scale were significantly stronger than those with the Extrapunitive/Manipulative Motivations scale.

Finally, an fMRI study (15) aimed at investigating the neural correlates of suicide and its main risk factor (i.e., mental pain), has underlined a general deactivation in frontal cortical areas of suicide attempters (i.e., BA 46, 10, and 6), namely with a reduced activation in the left dorsolateral prefrontal cortex (BA 46), in the right anterior prefrontal cortex (BA 10), and the left medial prefrontal cortex (BA 6).

### Mental Pain and Suicide in Samples of Students

Several studies, conducted with university students, have demonstrated associations between psychache and a history of suicidal ideation (42) and suicide attempts (43) as well as the role of psychological pain in the prediction of suicidal manifestations over and above depression, hopelessness (13, 44, 45), and self-destructive criterion (46). Table 3 presents reviewed studies of this category. By confirming these results in a large Chinese students sample, You et al. (47) reported that psychache and life satisfaction were both significant predictors for suicidal ideation and suicide attempt. Specifically, psychache had stronger power in predicting suicidal ideation and suicide attempt than life satisfaction. However, for suicidal ideation, life satisfaction, regardless of psychache, has a significant predictive role. That is, psychache partially mediated the relationship between life satisfaction and suicidal ideation. These findings indicate that life satisfaction may relieve the psychache and, therefore, reduces the risk for suicidal ideation and suicide attempt.

**Table 3:** **Studies on mental pain and suicide in samples of students**.

Study	Title	Aim	Sample information	Measure of mental pain	Measure of suicide
Campos et al. (49)	Self-report depressive symptoms do not directly predict suicidality in non-clinical individuals: contributions toward a more psychosocial approach to suicide risk	To use a longitudinal design to test several hypotheses. Study 2 assessed the hypothesis that change in suicide ideation is associated with change in psychache after controlling for changes in depression and hopelessness. Study 3 tested the hypothesis that the combination of psychache and hopelessness fully mediated the relationship between depression and life-time suicidality, and that hopelessness related indirectly to life-time suicidality through psychache	Sample size: S2 90 undergraduate students having a history of suicidal ideation or suicide attempt; S3 280 university students Mean age: S2 = 18.31 (SD = 2.24); S3 = 19.73 (SD = 2.17) Female: S2 = 87%; S3 = 70%	PAS	BSS SBQ-R
DeLisle and Holden (44)	Differentiating between depression, hopelessness, and psychache in university undergraduates	To measure the overlap between depression, hopelessness, and psychache constructs in predicting suicide risk	Sample size: 587 undergraduate students Mean age: 18.72 years (SD = 2.49) Female: 78%	PAS	BSS RASQ
Flamenbaum and Holden (7)	Psychache as a mediator in the relationship between perfectionism and suicidality	To assess whether psychache mediates the relationship between perfectionism and suicide	Sample size: 264 university students Mean age: 18.91 (SD = 3.34) years Female: 75.8%	PAS	Five items assessing suicide history BSS RASQ
Holden et al. (46)	Development and preliminary validation of a scale of psychache	To assess psychometric properties of the Psychache Scale and its association with suicidal manifestations	Sample size: S1 = 294 university students; S2 = 211 university students Mean age: S1 = 19.1 (SD = 1.6); S1 = 19.4 (SD = 2.4) Female: S1 = 76%; S2 = 100%	PAS	RASQ SMQ
Leenars and Lester (43)	A note on Shneidman’s Psychological Pain Assessment Scale	To explore validity and reliability of the PPAS as a correlate of suicidality	Sample size: 127 undergraduate students Mean age: 22.90 (SD = 6.40) years Female: 71%	PPAS	Questions about prior suicidal ideation, prior suicide attempts, and lethality of prior attempts
Lester (42)	Psychache, depression, and personality	To explore the correlation of psychache with a history of suicidal ideation and suicide attempts (and manic-depressive tendencies and temperament)	Sample size: 51 undergraduate students Mean age: 24.8 (SD = 7.1) years Female: 76%	PPAS	Questions assessing history of suicidal ideation, and history of suicide attempts
Troister et al. (19)	A 5-month longitudinal study of psychache and suicide ideation: replication in general and high-risk university students	To evaluate whether psychache and suicidality are associated, and whether this association continues when other suicide-relevant variables of depression and hopelessness are controlled statistically	Sample size: 945 university students into two groups; G1, 683 general sample of participants; G2, 262 high-risk university students Mean age: G1 = 18.23 (SD = NA) years; G2 = 18.17 (SD = NA) Female: G1 = 80%, G2 = 80%	PAS	Five questions asked about lifetime suicide attempts BSS
Troister and Holden (13)	Factorial differentiation among depression, hopelessness, and psychache in statistically predicting suicidality	To evaluate the unique contributions of psychache, depression, and hopelessness in the prediction of suicide ideation	Sample size: 2,974 university students Mean age: 18.31 (SD = 2.26) years Female: 71.8%	PAS	BSS
Troister and Holden (48)	A two-year prospective study of psychache and its relationship to suicidality among high-risk undergraduates	To use a longitudinal design to investigate psychache contribution to suicidality in at-risk university students	Sample size: 41 at-risk university students Mean age: 17.95 (SD = 0.95) years Female: 83%	PAS	BSS
Troister et al. (45)	Comparing psychache, depression, and hopelessness in their associations with suicidality: a test of Shneidman’s theory of suicide	To test Shneidman’s theory of suicide by evaluating the contributions of psychache, depression, and hopelessness, to the statistical prediction of suicidality	Sample size: 1475 undergraduate students Mean age: (18.36, SD = 2.09) Female: 71%	PAS	Five questions asked about lifetime suicide attempts BSS
You et al. (47)	Effects of life satisfaction and psychache on risk for suicidal behavior: a cross-sectional study based on data from Chinese undergraduates	To investigate the predictive power of life satisfaction and psychache on risks for suicidal ideation and suicide attempt in Chinese university students	Sample size: 5988 college students Mean age: 19.94 (SD = 1.38) Female: 46%	PAS	Two questions assessing suicidal ideation Three questions assessing suicide attempt

*NA, not available; S1, Study 1; S2, Study 2; S3, Study 3*.

*BSS, Beck Scale for Suicide Ideation; PAS, Psychache Scale; PPAS, Psychological Pain Assessment Scale; RASQ, Reasons for Attempting Suicide Questionnaire; SBQ-R, Suicide Behaviors Questionnaire-Revised; SMQ, Suicidal Manifestations Questionnaire*.

Flamenbaum and Holden (7), using a structural equation model, determined that psychache fully mediated the relationship between perfectionism and suicidality.

When longitudinally evaluating the relationship between psychache and suicidality, Troister and Holden (48) have confirmed the specific contribution of psychological pain as a predictor of suicidal ideation by demonstrating that modification in psychache contributed unique variance predicting change in suicide ideation both at baseline and at 2-year follow-up. In line with their previous research studies, Troister et al. (19) have revealed that psychological pain was significantly associated with the total suicide ideation, motivation, and preparation, by further confirming the same results at 5-month follow-up. Furthermore, associations between psychache and total suicide ideation, motivation, and preparation remain statistically significant after controlling for depression and hopelessness. The last interesting result of this study was that variation of the levels of psychological pain was significantly related to change in each of total suicide ideation. A recent paper (49) confirmed this evidence finding that 3-year changes in suicide motivation and preparation were significantly associated with changes in psychache but not to changes in depression or hopelessness. In the same article (49), a disclosure of the association between psychological pain, depressive symptoms, hopelessness, and suicidality was outlined. Results showed that depressive symptoms related to life-time suicidality through hopelessness and psychache. Hopelessness connected indirectly with life-time suicidality through psychache that related directly with life-time suicidality.

### Mental Pain and Suicide in Samples of Special Populations

The relationship between mental pain and suicide was further addressed in special populations of prisoner, homeless, and soldiers (Table 4).

**Table 4:** **Studies on mental pain and suicide in samples of special populations**.

Study	Title	Aim	Sample information	Measure of mental pain	Measure of suicide
Coohey et al. (52)	Sources of psychological pain and suicidal thoughts among homeless adults	To assess the association between several sources of psychological pain (i.e., drug problems, family relationship problems, social problems, psychiatric problems, past emotional abuse, past physical abuse, and past sexual abuse) and suicidal thoughts	Sample size: 457 homeless adults Mean age: 38.7 years (SD = NA) Female: 62%	ASI questions on sources of psychological pain	ASI questions about current suicidal thoughts and past suicide attempts
Mills et al. (50)	An evaluation of the Psychache Scale on an offender population	To test the hypothesis that the Psychache Scale is more strongly related to suicide indicators than measures of either depression or hopelessness in an offender population	Sample size: 136 inmates of a medium security prison Mean age: 38.0 (SD = 11.0) years Female: 0%	PAS	DHS
Patterson et al. (53)	Psychache and suicide ideation among men who Are homeless: a test of Shneidman’s model	To evaluate psychache as a stronger predictor of suicide among homeless	Sample size: 97 homeless Mean age: 46.58 (SD = 11.97) years Female: 0%	PAS	BSS Questions about history of a previous suicide attempt and the lifetime number of suicide attempts
Pereira et al. (51)	Testing Shneidman’s model of suicidality in incarcerated offenders and in undergraduates	To evaluate psychache as a stronger predictor of suicide among offenders and undergraduates	Sample size: 233 participants divided into three groups; G1, incarcerated offenders (73); G2, male undergraduate students (80); G3, female undergraduate students (80) Mean age: G1 = 44.89 (SD = 9.94) years, G2 = 19.04 (SD = 1.62) years, G3 = 19.55 (SD = 1.63) years Female: G1 = 0%, G2 = 0%, G3 = 100%	PAS	DHS
Shelef et al. (54)	Emotional regulation of mental pain as moderator of suicidal ideation in military settings	To examine how mental pain and emotional regulation of mental pain contribute to suicide ideation. Additionally, it explores whether emotional regulation of mental pain moderates the relationship between mental pain and suicide ideation.	Sample size: 168 soldiers divided into three groups; G1, soldiers attempted suicide (58); G2, soldiers psychologically treated without a history of suicide attempt (58); G3 soldiers control group (50) Mean age: total sample 19.7 (SD = 1) years, data for single groups NA Female: total sample = 40.5%, G1 = 38.0%, G2 = 39.7%, G3 = 42.0%	OMMP TMPS	SSI

*NA, not available; G1, Group 1; G2, Group 2; G3, Group 3*.

*ASI, Addiction Severity Index; BSS, Beck Scale for Suicide Ideation; DHS, Depression, Hopelessness and Suicide Screening Form; OMMP, Orbach and Mikulincer Mental Pain Scale; PAS, Psychache Scale; SSI, Scale for Suicide Ideation; TMPS, Tolerance for Mental Pain Scale*.

Two studies were founded in prisoner populations. Mills et al. (50) found a significant association between the score of the Psychache Scale and a history of prior suicide attempts. A study by Pereira et al. (51) confirmed the significant correlation between variables (with a large effect size), and it showed that psychache was the more significant statistical predictor of self-harming ideation and act than either depression and hopelessness.

Assessing the mental pain contribution to suicidality among homeless, a research study of Coohey et al. (52) showed that psychological pain was related to thoughts of suicide, by underlining that homeless with suicidal thoughts reported more psychological pain than homeless without suicidal thoughts. Furthermore, when considering each additional source of psychological pain, participants were 46% more likely to show suicidal thoughts supporting the authors’ hypotheses that the number of sources of psychache explained suicidal thoughts above and beyond other important predictors. In line with these results, Patterson and Holden (53) further confirmed the Shneidman’s theory of suicidality by indicating the associations between psychache and suicide factors (i.e., ideation, preparation, attempt history, as well as number of lifetime attempts) as having the higher correlation values than depression, hopelessness, and life meaning. Moreover, the authors (53) have also confirmed, by using multiple regression analyses, that psychological pain was the only significant factor predicting unique variance over and above the contribution by depression, hopelessness, and life meaning.

Meaningful results were recently reported by Shelef and Holden (54) testing a population of 168 soldiers. The major result comprises the evidence that emotional regulation of mental pain moderates the association between psychological pain and suicide ideation. In other terms, the authors (54) showed that mental pain was related to higher suicidal ideation only among soldiers who have difficulty to regulate mental pain.

## Discussion

Intense negative emotions, such as guilt, shame, and hopelessness may become a generalized experience of unbearable mental pain, especially when there is no foreseeable change in the future. Consequently, individuals may seek to escape their “psychache” dying by suicide (8). Several theories, measurements, clinical risk assessments, and studies have been developed over the past decade in order to describe, assess, and confirm mental pain as a central key in suicide [e.g., Ref. (8, 46, 55)]. Mental pain has been shown in the literature as having a strong association with suicidality and it seems to be caused by the basic psychological needs of the individual (e.g., love, closeness, appreciation, and independence) not being sufficiently satisfied. By following a recent editorial from Tossani (4), it is highly significant from a clinical point of view to improve the study of the main implications linking mental pain to higher suicidal risk. That is, it is clinically important to assess psychological pain because it may be conceptualized as the core clinical factor that leads individuals to be vulnerable for suicidality. Therefore, when taking the potential clinical implications related to this relatively novel concept into account, to the very best of our knowledge, the present study is the first review manuscript aimed at systematically investigating the published original research reports evaluating the emerging clinical links between psychological pain and suicidal factors.

Associations between mental pain and suicidality were found in patients with mood disorders, in other clinical and non-clinical samples. High levels of mental pain may represent a condition of vulnerability to suicidal ideation, suicidal attempts, and suicide acts. When taking the clinical consequences of the research studies analyzing the relationship between psychache and suicide into consideration, the findings revealed the unique value of psychological pain in predicting suicidal behavior (i.e., ideation, attempts, and acts) when controlling for the effects of other variables (i.e., depression, hopelessness, aggression-impulsivity) potentially associated with suicidality (21). In other terms, the results indicate that levels of mental pain are associated with an increased risk of suicide, independently from the severity of depressive condition (25). Research findings confirmed that mental pain is much more than the sum of negative feelings and sensations, making it a uniquely intolerable experience (29). It may be accompanied by the belief that it is not possible to change and this may lead to the conclusion that the only solution is self-destruction (29). Recent studies have shown that mental pain alone will not lead to suicidal behavior, but will become critical when the person has no ability to regulate the emotional pain experienced. That is, if mental pain is controlled, the single effect of many factors would be largely attenuated or become insignificant. Recent studies have showed that mental pain alone did not significantly predict suicide lethality [e.g., Ref. (2, 32)]. When mental pain is associated with inability to communicate the stress, painful feelings remain unaddressed, help is not available, and more serious forms of suicidal behavior might ensue (32, 33). The lethality and seriousness of suicidal behavior were strongly predicted by the interaction of mental pain with other variables, such as interpersonal and communication difficulties (33), schizoid traits (2), alexithymia, and loneliness (32). These associations are very meaningful from a clinical point of view. The factor that plays an important role in the decision to attempt suicide consists of the degree to which an individual can tolerate own mental pain that in turn depends from own personal and social resources. Literature highlighted that lack of individual resources, as expressed by an insecure attachment style (35) or an inability to disclose emotions and thoughts (2, 32, 33, 36) can amplify mental pain eliciting an intolerable experience that facilitates lethal suicidal actions. Some authors (40) suggested that when mental pain is experienced as unbearable and persistent, a reduced awareness of the body and its signals increases the likelihood to perceive it as an object as well as an easier target to attack.

Recent studies have examined the mediation role of mental pain, finding that this construct mediates both the relationship between self-destruction and suicidal tendency (11), and the association between depression and suicide risk (39).

Longitudinal studies confirmed the critical role of mental pain in student populations, demonstrating that changes in suicide motivation and preparation were significantly associated with changes in psychache (19, 52, 53). However, to further support these results, additional longitudinal studies with clinical samples are needed.

By supporting the evidence that psychological pain and its different dimensions (i.e., pain avoidance, emotional pain) play a central role in predicting suicide, the results of this review study could further underline the clinical relevance of psychache in prevention (i.e., early detection) as well as in treatment of suicide. In this regard, mental pain may represent, from a clinical point of view, an important therapeutic target, when considering that diminishing levels of psychache potentially means to decrease the risk of suicidal acts (22). Therefore, by identifying psychological pain in order to evaluate the suicidal risk, psychache can be targeted during clinical interventions and considered amenable to treatment (45, 55). It is also suggested that suicide is a balanced consequence, depending on the presence of risk factors and the absence of protective factors against suicide. For example, if psychache is balanced by the presence of protective factors, such as life satisfaction, the unbearable mental pain can become acceptable and the individual would stay to live on (47). Further research into positive variables is needed.

In conclusion, several measures have been developed to assess mental pain (4) and these instruments can be easily included into risk assessment along with measures of depression and hopelessness, in order to improve the accuracy of suicide risk prediction.

According to recent developments on clinical psychometrics (56), clinimetrics provides significant possibilities for evaluating clinical variables, such as mental pain (4, 57).

Based on this background, it is highly meaningful to clinimetrically evaluate measurement aspects (i.e., construct validity) of the instruments in order to assess psychache in clinical setting.

## Author Contributions

All authors participated in the concept and writing of this manuscript. All authors approved the final version of the manuscript.

## Conflict of Interest Statement

There are no potential conflicts of interest or any financial or personal relationships with other people or organizations that could inappropriately bias conduct and findings of this study.
